# A proof-of principal study using phase-contrast imaging for the detection of large airway pathologies after lung transplantation

**DOI:** 10.1038/s41598-020-75185-4

**Published:** 2020-10-28

**Authors:** Stephan Umkehrer, Carmela Morrone, Julien Dinkel, Laura Aigner, Maximilian F. Reiser, Julia Herzen, Ali Ö. Yildirim, Franz Pfeiffer, Katharina Hellbach

**Affiliations:** 1 Chair of Biomedical Physics, Physics Department & Munich School of BioEngineering, Technical University of Munich (TUM), Garching, Germany; 2 Member of the German Center for Lung Research (DZL), Comprehensive Pneumology Center Munich (CPC-M), Ludwig-Maximilians University Munich, Munich, Germany; 3 Department of Radiology, University Hospital, Ludwig-Maximilians University Munich, Munich, Germany; 4 Institute of Lung Biology and Disease, Helmholtz Zentrum München, Neuherberg, Germany; 5 Department of Diagnostic and Interventional Radiology, School of Medicine & Klinikum rechts der Isar, Technical University of Munich, Munich, Germany; 6 Department of Diagnostic and Interventional Radiology, University Hospital of Heidelberg, Ruprecht-Karls-University Heidelberg, Heidelberg, Germany; 7 Translational Lung Research Center Heidelberg (TLRC), Ruprecht-Karls-University Heidelberg, German Center for Lung Research (DZL), Heidelberg, Germany

**Keywords:** Biophysics, Physiology, Anatomy, Medical research, Signs and symptoms, Physics

## Abstract

In this study we aim to evaluate the assessment of bronchial pathologies in a murine model of lung transplantation with grating-based X-ray interferometry in vivo. Imaging was performed using a dedicated grating-based small-animal X-ray dark-field and phase-contrast scanner. While the contrast modality of the dark-field signal already showed several promising applications for diagnosing various types of pulmonary diseases, the phase-shifting contrast mechanism of the phase contrast has not yet been evaluated in vivo. For this purpose, qualitative analysis of phase-contrast images was performed and revealed pathologies due to previous lung transplantation, such as unilateral bronchial stenosis or bronchial truncation. Dependent lung parenchyma showed a strong loss in dark-field and absorption signal intensity, possibly caused by several post transplantational pathologies such as atelectasis, pleural effusion, or pulmonary infiltrates. With this study, we are able to show that bronchial pathologies can be visualized in vivo using conventional X-ray imaging when phase-contrast information is analysed. Absorption and dark-field images can be used to quantify the severity of lack of ventilation in the affected lung.

## Introduction

Lung transplantation often remains as the last therapeutic option in advanced pulmonary disease such as mucoviscidosis, COPD or idiopathic pulmonary fibrosis, especially when conservative therapeutic approaches have not been successful. The International Society for Heart and Lung Transplantation reported a total number of 4452 lung transplantations that were performed in 2017^1^. The mean survival after a primary lung transplant procedure between 2010 and June 2017 was 6.7 years. Surviving the first year after transplantation increased the median survival to 8.9 years. The most common causes of death after transplantation are infection, graft failure and acute rejection as well as bronchiolitis obliterans syndrome (BOS)^1^. Apart from changes occurring directly after surgery like a pneumo- or hemothorax, complications affecting the airways, mainly in the region of the anastomosis, are common post-transplantational risks. The most frequent airway complication besides anastomosis dehiscence, infections, or fistula is bronchial stenosis with a frequency ranging between 3.5 and 32%^2–5^. The stenosis occurs either in the area of the anastomosis or slightly further distal^3^. Bronchial stenosis may become symptomatic with cough, dyspnoea or recurrent pneumonia. A decline in lung function may also be observed. As the clinical symptoms of bronchial stenosis are ambiguous, bronchoscopy—as an invasive examination technique—is the standard of reference for the diagnosis of this pathology^6^. Chest X-ray may indicate bronchial stenosis by showing consecutively hypoventilated areas. To actually verify the luminal constriction itself computed tomography (CT) imaging is necessary, coming along with a higher radiation dose.

Additional to conventional attenuation-based X-ray radiography, a method creating phase-contrast and dark-field images by using the refraction and small-angle scattering of the X-ray beam has been developed. Therefore, a three-grating Talbot-Lau interferometer is interposed between the X-ray source and the detector^7,8^. The diagnostic value of dark-field imaging (DFI) has already been proven for the diagnosis of several pulmonary diseases, such as pulmonary fibrosis, emphysema, pneumothoraces and lung cancer in mouse models^9–12^. For these approaches alterations in the high dark-field signal of healthy lung parenchyma, caused by small angle X-ray scattering at alveolar walls, were used to detect disease-related changes in lung tissue, indicated by a loss of dark-field signal strength.

In contrast to DFI, phase-contrast imaging (PCI) using a conventional X-ray source has not been evaluated for examination of the lungs yet. It is based on the phase-shift of the X-ray beam when passing through tissue, leading to a visual impression of enhanced borders between different anatomical structures, such as the trachea or main bronchi^13^.

In this in vivo proof-of-principle study, PCI was assessed for the first time with regard to its use for bronchial imaging in mice. We assumed that PCI would be an appropriate method to visualize the bronchial system due to the interface between the air-filled bronchi and its surrounding tissue. Furthermore, we hypothesized that visualizing bronchial pathologies in mice that underwent single lung transplantation would be possible for the first time using radiography acquired with grating-based interferometry.

## Results

### Quantitative image analysis—absorption and dark-field signal measurements of lung parenchyma

**Figure 1:**
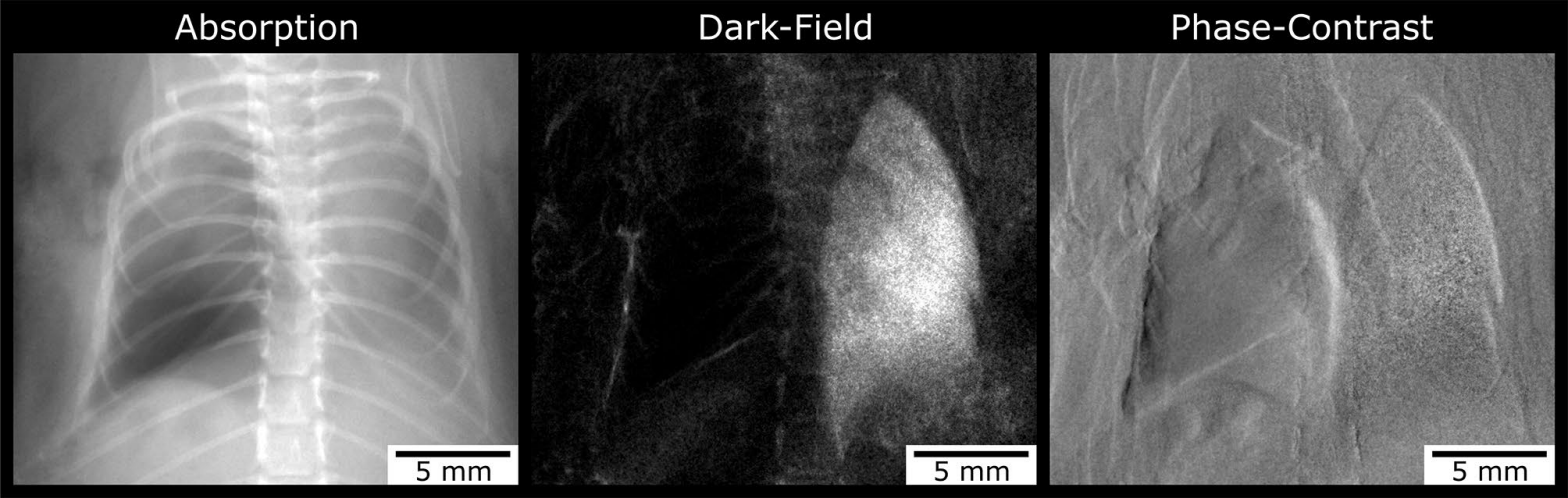
Pneumothorax. Example of one out of four mice that presented with a unilateral, left-sided pneumothorax after transplantation. The pneumothorax is indicated by hyper-transparency in the absorption image (left side). There is no signal observed in the corresponding dark-field image (middle).

The lungs of 46 transplanted mice were analysed for quantitative absorption and dark-field signal intensities at TP1. Four mice had to be excluded from the measurements due to the development of a left-sided pneumothorax (Fig. 1). One mouse died shortly after the first scan and therefore was excluded as well.

**Figure 2:**
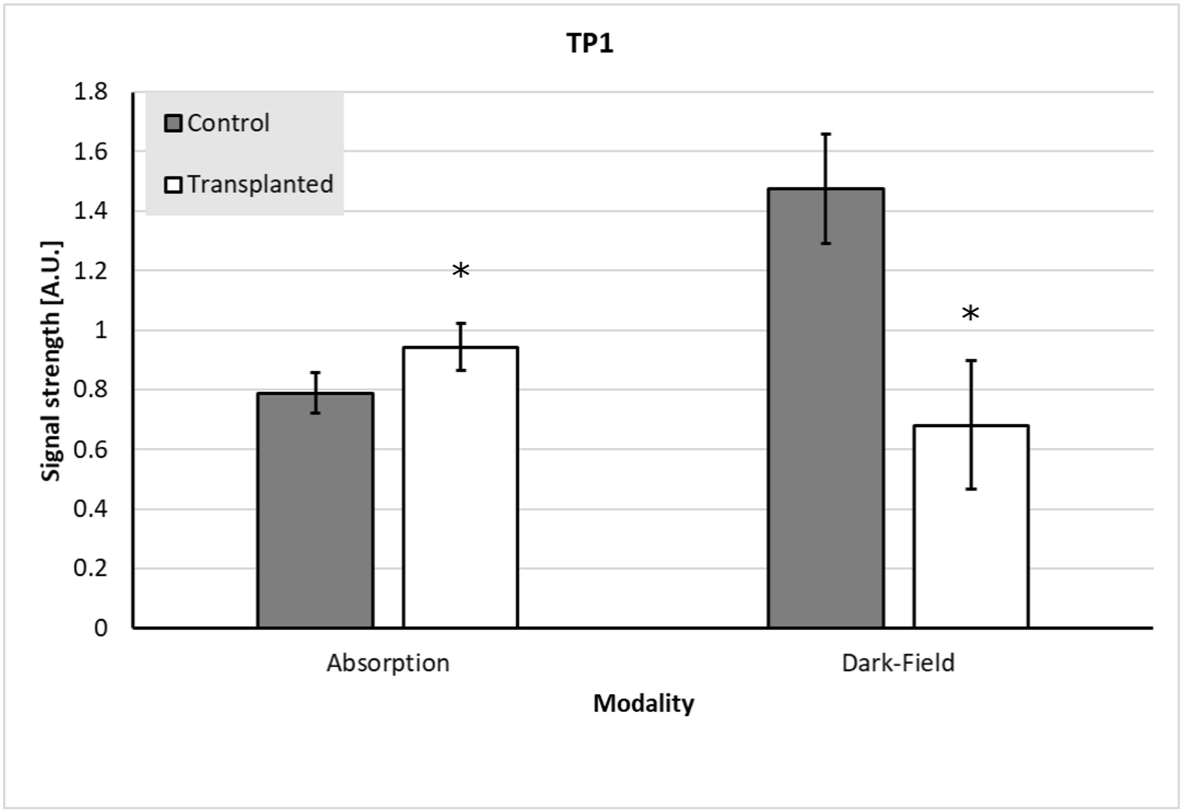
Comparison of transplanted with control lungs. When comparing the transplanted lungs (white bars) with the control lungs (grey bars) at TP1, a significant increase in absorption and decrease in dark-field signal intensities of the transplanted lungs could be observed. The error bars indicate the standard deviation of the respective signal of lungs, the asterisks indicate the significance.

When comparing the dark-field signal intensities of the transplanted ($0.681 \pm 0.215$) with the control lungs ($1.475 \pm 0.184$), a significant loss of signal strength of the transplanted lungs was observed ($p < 0.001$). For absorption signal intensity, a significant increase of absorption between the transplanted ($0.943 \pm 0.078$) and control lungs ($0.788 \pm 0.069$) was measured ($p < 0.001$) (Fig. 2).

**Figure 3:**
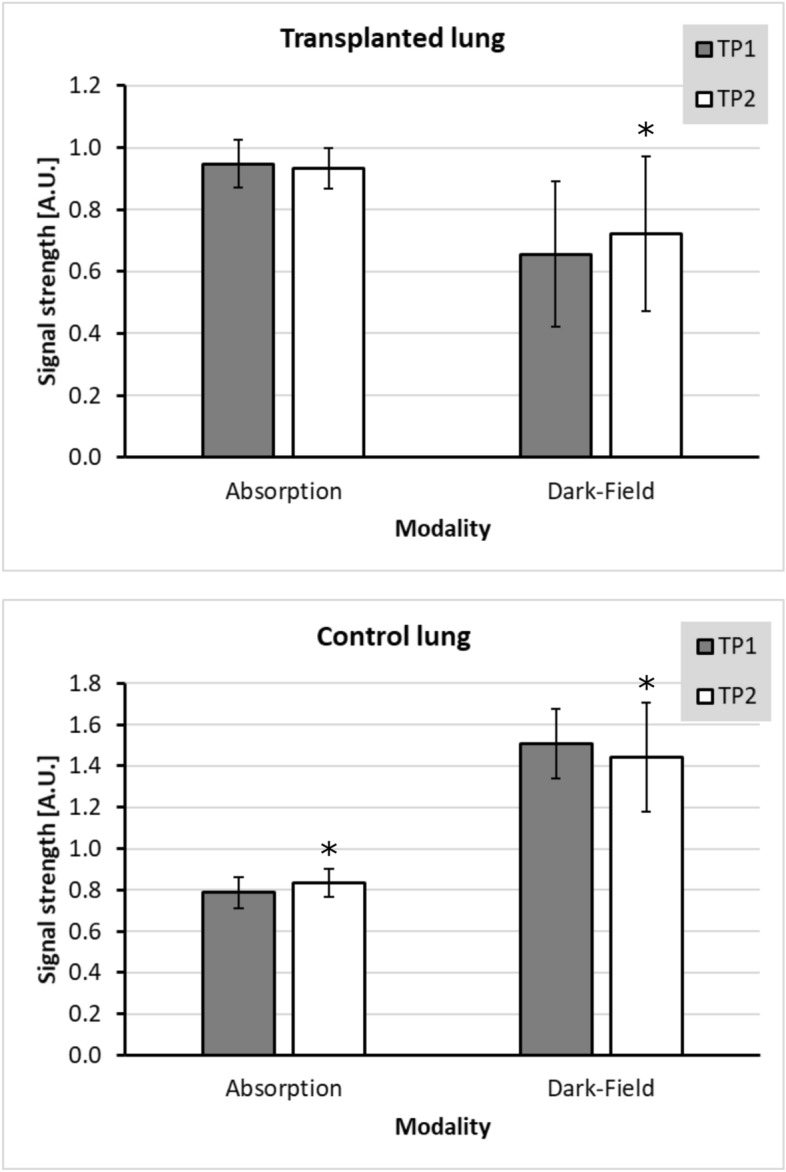
Development of signal intensities over time. When analysing the signal intensities of the transplanted lungs, a slight but not yet significant decrease of absorption was found between TP1 (grey bars) and TP2 (white bars) ($p = 0.09$). Dark-field signal showed a slight, statistically significant increase comparing TP2 with TP1 ($*p = 0.04$). Control lungs showed a statistically significant increase in absorption and decrease in dark-field signal intensities between TP2 and TP1 ($*p < 0.05$). The error bars indicate the standard deviation of the respective signal of lungs, the asterisks indicate the significance.

As a second approach for quantitative analysis, the signals derived by the lungs over time, comparing TP1 and TP2, were measured. Therefore, the lungs of a total of 27 mice, that underwent a second measurement, were analysed. There was no significant change in absorption signal intensity for the transplanted lungs, comparing TP1 and TP2 ($p = 0.09$). By contrast, a slight but still significant increase ($p = 0.04$) of dark-field signal intensity for the transplanted lungs was observed between TP1 ($0.656 \pm 0.234$) and TP2 ($0.722 \pm 0.251$). Interestingly, when taking a look at the control lungs, a significant decrease in dark-field signal intensity (TP1: $1.507 \pm 0.168$; TP2: $1.442 \pm 0.263$; $p < 0.05$) as well as increase of absorption in absorption images (TP1: $0.789 \pm 0.075$; TP2: $0.836 \pm 0.067$; $p < 0.05$) could be observed (Fig. 3).

### Qualitative image analysis—phase contrast imaging of bronchial stenosis

**Figure 4:**
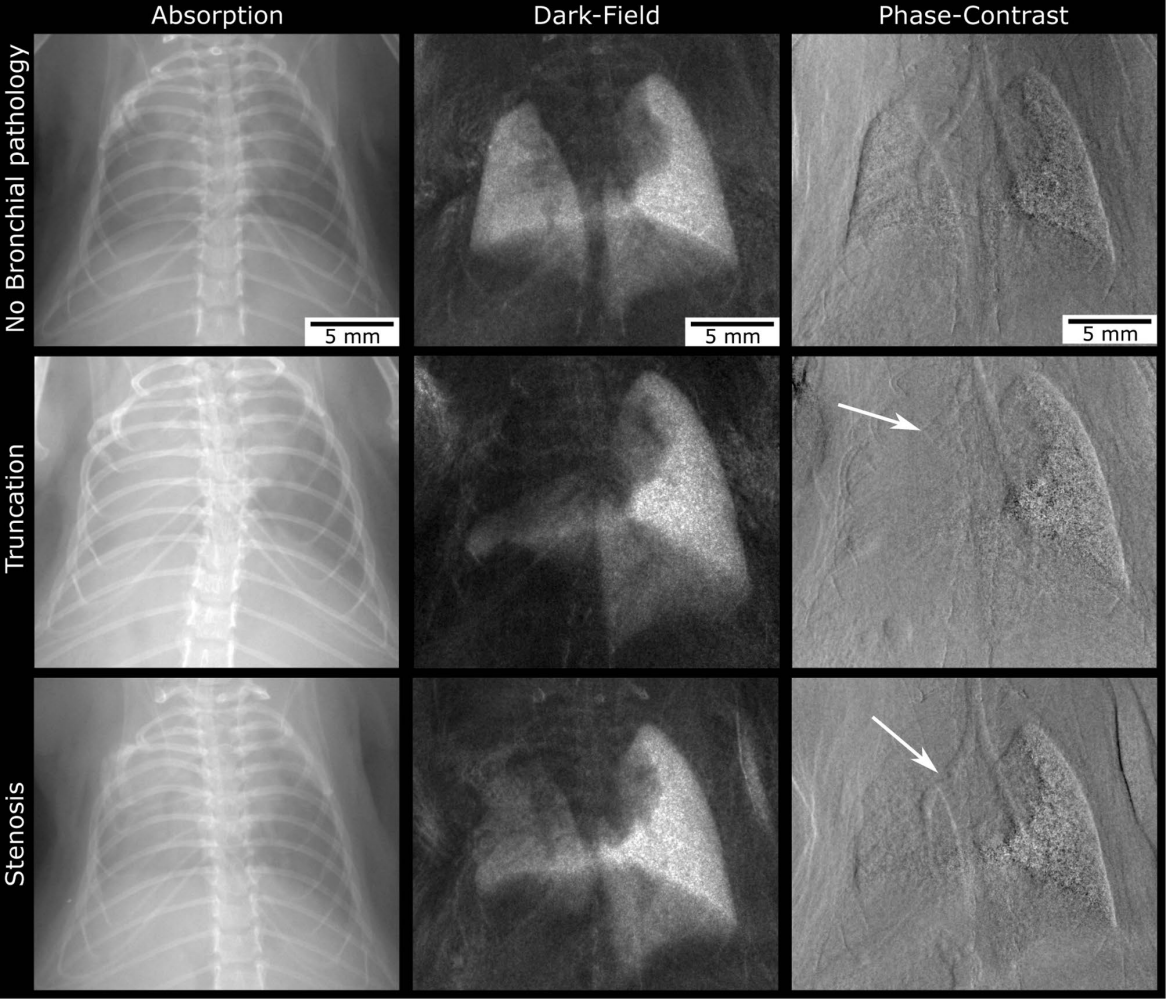
Bronchial pathologies. Mouse after left-sided lung transplantation without visible bronchial pathology in the phase-contrast image (upper row). Note that the dark-field as well as the absorption image show good ventilation of the transplanted lung. By contrast, the left main bronchus distal to the cuff is not visible on the phase-contrast image from the mouse below (arrow; middle row), consistent with bronchial truncation. The corresponding dark-field and absorption image of this mouse show total atelectasis of the upper and middle parts of the transplanted lung. In case of the third mouse (lowest row), the lumen of the bronchus narrows at the level of the cuff (PCI; arrow). In contrast to truncation, the more distal parts of the bronchus remain visible, indicating stenosis. Ventilation of the left lung is constrained (dark-field image) but not as severe as in case of truncation.

When carefully analysing the phase-contrast images of the transplanted mice at TP1, 24 lungs out of 46 lungs showed decreased ventilation, of which $n = 14$ had visible irregularities of the left main bronchus. These irregularities either became visible as a stenosis ($n = 4$) of the bronchus or as a bronchial truncation ($n = 10$) (Fig. 4).

**Figure 5:**
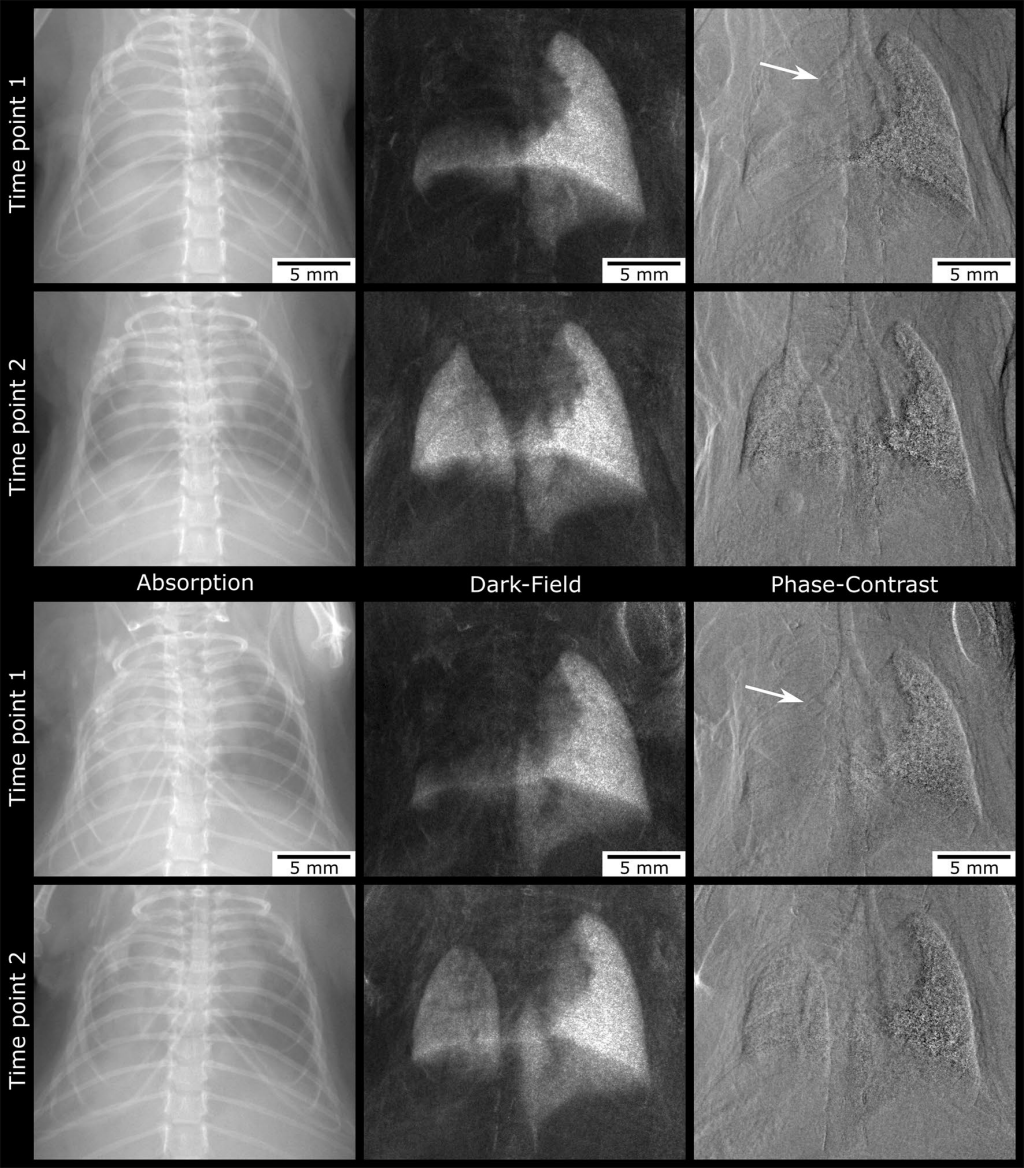
Two examples for decreasing bronchial truncation over time. A loss of ventilation can be observed in the absorption and more clearly in the dark-field image at time point 1. The corresponding phase-contrast image shows bronchial truncation at the level of the cuff. Two months later (time point 2) ventilation of the transplanted lung has increased. Now, the left main bronchus is continuously visible as the phase-contrast image reveals.

**Figure 6:**
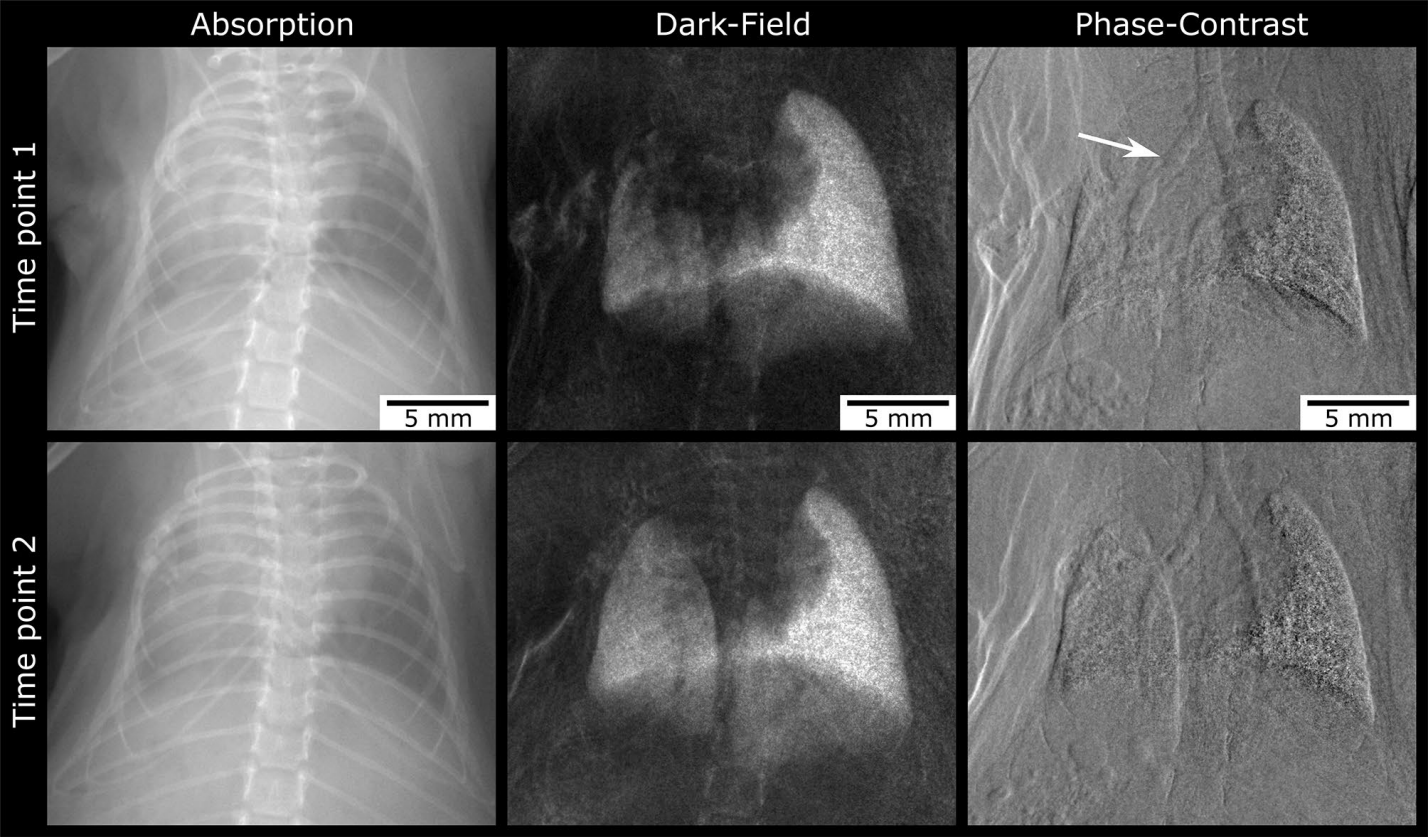
Decreasing bronchial stenosis over time. At time point 1 stenosis of the main bronchus at the level of the cuff can be observed in the phase-contrast image. Interestingly, the bronchus distal of the cuff is slightly dilated (1.5 mm). Two months later (time point 2) there is no stenosis of the bronchus visible anymore. The lumen of the bronchus distal the cuff has normalized (1.1 mm). Corresponding absorption and dark-field images show slightly improved ventilation of the left lung compared to time point 1.

A total of 8 mice showed improved ventilation of their transplanted lungs in absorption and DFI at TP2, compared to TP1. When taking a closer look at these animals, a decrease of left-sided bronchial irregularities over time could be observed in 6 mice: either were the bronchial parts located distally the cuff visible again or was the degree of stenosis declining (Figs. 5 and 6).

## Discussion

Besides primary graft dysfunction, acute rejection, postoperative infection and haemorrhage, as well as vascular anastomotic complications, large airway complications are one of the most important adverse events after lung transplantation^14^. The most important cause for large airway complications is central airway stenosis (CAS). It can include the anastomosis region. Up to 15% of all patients develop CAS within one year after lung transplantation^15^. Complications involve bronchial dehiscence, exophytic excessive granulation tissue formation, tracheo-bronchomalacia, bronchial fistulas, and endobronchial infections^2^. Most of these complications, including CAS, can be visualised using computed tomography^16^. By contrast, conventional absorption imaging shows alterations due to indirect changes of bronchial pathologies, such as atelectasis, rather than visualizing the bronchial system itself. Therefore, the authors of this study hypothesised phase-contrast imaging to be a helpful tool for diagnosing post transplantational large airway complications, as it strongly enhances the edges of the bronchial system, making it visible with a conventional imaging setup for the first time.

We were able to clearly distinguish bronchial stenosis—visible as decreasingly aerated bronchial lumen, followed by dystelectasis of the dependent lung parenchyma—from bronchial truncation, where there was no aerated bronchial lumen visible anymore, leading to distinct ventilation deficiency of the affected lung. Alterations of bronchial pathologies over time were also visible, helping to understand, how pulmonary atelectases altered. This might be very helpful when thinking about clinical practice, as targeted diagnostic and therapeutical bronchoscopy can be performed in these patients, even without the need to perform a CT scan prior to intervention.

When analyzing the signals derived by the transplanted lungs and comparing it with those of the control lungs, a significant change in both dark-field and absorption images was detected after lung transplantation. Absorption images showed an increase of absorption, and the corresponding dark-field images revealed a loss of signal intensity, both being consistent with a decreased proportion of ventilated pulmonary parenchyma within the transplanted lung. This might be caused by several post transplantational pathologies, such as atelectasis due to bronchial stenosis, pleural effusion and/or haemorrhage, as well as consolidations due to pulmonary infiltrates (e.g. infectious, necrotic or inflammatory). As there were no CT scans acquired in this study, this remains unclear. It is important to point out, that the changes in signal intensities observed in the transplanted lungs are not necessarily connected to large airway pathologies, as complications are diverse and multiple pathologic changes can occur simultaneously: The most important postoperative complications in animal studies besides large airway pathologies include thrombosis of a bronchial vein or artery, respiratory infections, pleural effusion, pulmonary edema, and graft rejection. This might explain the observation, that not all of the lungs showing a loss of ventilation presented visible bronchial pathologies^17,18^.

After two months (TP2) an overall increase of dark-field signal intensity was measured, indicating an improved ventilation of the transplanted lungs compared to TP1. Although no significant change of absorption signal intensity was detected, a slight decrease of absorption was observed, which can be explained by the better aeration of the transplanted lungs as well. In the corresponding phase-contrast images, some of the lungs showing signal alterations as described, revealed recurrent bronchial stenosis, most likely being the reason for improved ventilation. Reversible bronchial stenosis can be caused by mucus plugging or bronchial haemorrhage. Development of scar tissue could rather be a reason for persisting bronchial stenosis, just as bronchial dehiscence, e.g. due to cuff displacement. According to literature, an undersized diameter of the cuff as well as foreign-body reactions due to the cuff may occur, possibly leading to bronchial stenosis and truncation. Furthermore, there is a given risk for twisting of the bronchi after anastomosis, also resulting in bronchial truncation^19^. Disadvantageously, for both time points in the experiments described in this work, the specific reason causing bronchial obstruction remains unclear, as we neither have histology from the cuff region nor CT scans. Besides, thinking from a more practical and diagnostic point of view, knowing the exact reason for a bronchial pathology might be less important than showing the pathology at all (and therefore making its diagnosis possible), as its cause will hardly be explained by conventional X-ray imaging alone. Further examinations based on CT and bronchoscopy would be necessary in any case. Nonetheless, investigations focusing on histopathological changes of the cuff region after lung transplantation are preferable.

When taking a look at the signal development over time of the control lungs, an interesting observation was made: Both absorption and dark-field images indicated signal changes similar to the transplanted lungs at TP1—to a very discrete but still significant extend. A possible explanation for this observation might be, that the control lungs had to overcompensate the poor condition of the transplanted lungs at TP1 and therefore were hyper-inflated. As the transplanted lungs slightly recovered over time, the recipients’ native lungs returned to their physiological state of capacity^20^.

Four animals had to be excluded from the signal analysis as they had developed a large left-sided pneumothorax after lung transplantation. According to literature, post transplantational pneumothoraces most likely occur due to bronchial leaks^21^. One major flaw of this study might be that there is no external standard of reference, as there are no computed tomography scans nor histological examinations of the large airways and the region of the cuff. The conventional X-ray images of the recipients’ own, right-sided lungs served as control, concerning the degree of ventilation as well as the bronchial system. This might be critical because—as shown for ventilation—the native organ can also be influenced by the transplantation. Further studies of the bronchial system with a strong standard of reference are necessary.

Multiple murine in vivo studies about imaging pulmonary diseases using grating-based radiography have been performed so far, almost unexceptionally focusing on the dark-field information and comparing it with absorption images. With the current study, we investigated the value of phase-contrast imaging for the first time, proving this imaging modality to markedly improve the diagnostic value of conventional chest radiography for the detection of large airway pathologies in living mice that underwent single lung transplantation. Additionally, this work perfectly shows the potential of grating-based X-ray imaging to address multiple organ-specific pathologies, as absorption (osseous, mediastinal structures), dark-field (lung parenchyma) and phase–contrast information (bronchial system) are simultaneously available. The authors of this study are fully aware of the fact, that this conventional imaging technique is most likely not going to replace CT or bronchoscopy in case of possible large airway pathologies. But thinking of future clinical applications, it might be eligible for earlier detection of bronchial stenosis and therefore help transferring the patient to dedicated airway diagnostics within a narrow time frame. However, the transition of this technique from pre-clinical research to clinical routine is far from being trivial. Different kinds of technical requirements have to be met, such as the increased field-of-view, the higher grating structures needed due to higher energies used, or the setup design. In order to investigate and address the technical challenges of transferring grating-based interferometry to humans, research has been extended to larger in vivo animals and post-mortem human in recent years^22–25^. Finally, the investigations on imaging have been successfully extended to human in the clinic at a prototype dark-field radiography setup where first studies are going on.

## Materials and methods

### Single left lung transplantation

All animal procedures were performed with permission of the local regulatory authority, Regierung von Oberbayern (ROB), Sachgebiet 54, 80534 München, approval number AZ 55.2-1-54-2532-61-2015. The ethics committee reviewed the application according to §15 TSchG German Animal Welfare Law.

A total of $n=102$ male mice were used for the experiments (all 8 to 12 weeks old), of which $n=51$ served as donors and $n=51$ served as recipients. Briefly, donors were anesthetized with an i.p. injection of ketamine/xylazine. The pulmonary artery (PA), bronchus and pulmonary vein (PV) were carefully separated one from the other with blunted forceps, prior to cuffing with, respectively, 24G, 20G and 22G cuffs. The left lung graft was perfused i.v. with $3\,$mL of Perfadex. The recipient mouse was anesthetized with a mixture of medetomidine ($1\,$mg/kg), midazolam ($0.05\,$mg/kg) and fentanyl ($0.02\,$mg/kg), intubated and connected to a small animal ventilator (Harvard apparatus), at a respiratory rate of $120\,$bpm and a tidal volume of $300\,$µL. The chest was opened on the left side between ribs 3 and 4 and the native left lung retracted with a clamp. The hilar structures were carefully separated one from the other with blunted forceps. After arrest of the blood and air flow towards the left lung, the cuffed graft PA, bronchus and PV were inserted into the recipient counterparts, and the connection between donor and recipient structures was secured with 9-0 sutures. The native left lung was removed and the incision in the chest was closed with a 6-0 suture, after removing all potential air bubbles from the chest. Antagonist was administrated and the animal was extubated when it showed signs of spontaneous breathing. After the operation, the recipient mice were allowed to recover at ${30}\,^{\circ }\hbox {C}$ and received buprenorphine ($0.1\,\hbox {mg}/\hbox {kg}$)^26^.

### Imaging protocol

All mice were imaged shortly after lung transplantation (time point 1/TP1). A subgroup of $n=27$ mice was scanned at a second time point two months later (TP2). The small-animal X-ray dark-field CT setup presented in our work was developed in collaboration with Bruker MicroCT and consists of a compact rotating CT gantry built into a conventional microCT housing, suitable for preclinical research^27,28^. The grating-interferometer was adapted to the gantry and consists of three gratings: a source grating (gold, period $p=10\,$µm, height $h=35\,$µm), a phase grating (nickel, period $p=3.24\,$µm, height $h=4\,$µm), and an analyser grating (gold, period $p=4.28\,$µm, height $h=45\,$µm). Different equipment in order to monitor physiological functions like breathing, heartbeat, and body temperature are implemented into the setup. The gantry system features a direct anode tungsten target X-ray tube (RTW, MCBM 65B-50 W) with a focal spot size of $50\,$µm $\times \, 50\,$µm, a Hamamatsu flat panel detector (C9312SK-06) with a gadolinium oxysulfide (GOS) scintillator and a pixel size of $50\,$µm $\times \, 50\,$µm. Due to magnification, the effective pixel size in the sample plane accounts to $29\,$µm $\times \, 29\,$µm. There was no filter in the beam.

During image acquisition the mice remained in supine position. Images were acquired using four stepping positions with an exposure time of 5 s per position. Reference images were acquired with the sample removed from the beam. The source was operated at 35 kVp and 20 W. The deposited dose during X-ray exposure results in $1.5\,$mGy. Subsequently, the images were processed by using an in-house Python script to calculate all three modalities from the images acquired during the phase-stepping procedure. A flow-chart of this study is shown in Supplementary Fig. 1.

### Quantitative image analysis

For quantitative signal analysis of the conventional absorption and the dark-field signal two masks were segmented for each mouse and measurement. Transplanted and native lung masks were segmented individually. The recipients’ native lungs, that did not undergo transplantation, served as control lungs in this study. The segmentation of the masks was performed by experienced radiologists (K.H., L.A.) on the basis of conventional absorption images using the software MATLAB. The masks included the free accessible lung parenchyma without the overlaying mediastinum and heart or the diaphragm. In order to increase the classification accuracy, osseous structures such as the ribs and the spine were excluded from the masks as well. The ribs and the spine were segmented automatically using binary erosion and dilation on pre-smoothed absorption images. An example for the choice of the ROIs is shown in Supplementary Fig. 2. A pixel per pixel comparison between absorption and dark-field images is possible as they result from the same exposure. The mean signal for the ROI within the mask was calculated.

### Qualitative image analysis

All images were assessed separately for the presence or absence of bronchial pathologies by two experienced lung radiologists (K.H., J.D.).

### Statistical analysis

To compare the dark-field and absorption signal intensities of the transplanted and control lungs, means and standard deviations of the signals were calculated. Means were tested for statistical significance using Student t-test for paired samples.

## Supplementary information

Supplementary Information.
