# Supplementary material for: A proof-of principal study using phase-contrast imaging for the detection of large airway pathologies after lung transplantation
Source: Sci Rep. 2020 Oct 28;10:18444. doi: 10.1038/s41598-020-75185-4 (PMC7595203; doi:10.1038/s41598-020-75185-4)
Supplement: Supplementary file 1 — Supplementary Information. [file 41598_2020_75185_MOESM1_ESM.pdf]

A Proof-of principal study using Phase-contrast imaging for thedetection of large airway pathologies after lung transplantation

Stephan Umkehrer[1,*], **Carmela Morrone**[2], **Julien Dinkel**[3,4], **Laura Aigner**[3], **Maximilian F. Reiser**[3,4], **Julia Herzen**[1], **Ali Ö. Yildirim**[2,4], **Franz Pfeiffer**[1,5,+], **and Katharina Hellbach**[3,6,7,+]

[1]Chair of Biomedical Physics, Physics Department & Munich School of BioEngineering, Technical University of Munich (TUM), Garching, Germany
[2]Comprehensive Pneumology Center Munich (CPC-M), Ludwig-Maximilians University Munich, Member of the German Center for Lung Research (DZL), Munich, Germany
[3]Department of Radiology, University Hospital, Ludwig-Maximilians University Munich, Munich, Germany
[4]Institute of Lung Biology and Disease, Helmholtz Zentrum München, Neuherberg, Germany
[5]Department of Diagnostic and Interventional Radiology, School of Medicine & Klinikum rechts der Isar, Technical University of Munich, Munich, Germany
[6]Department of Diagnostic and Interventional Radiology, University Hospital of Heidelberg, Ruprecht-Karls-University Heidelberg, Heidelberg, Germany
[7]Translational Lung Research Center Heidelberg (TLRC), Ruprecht-Karls-University Heidelberg, German Center for Lung Research (DZL), Heidelberg, Germany
[*]stephan.umkehrer@ph.tum.de
[+]These authors contributed equally to this work.

ABSTRACT

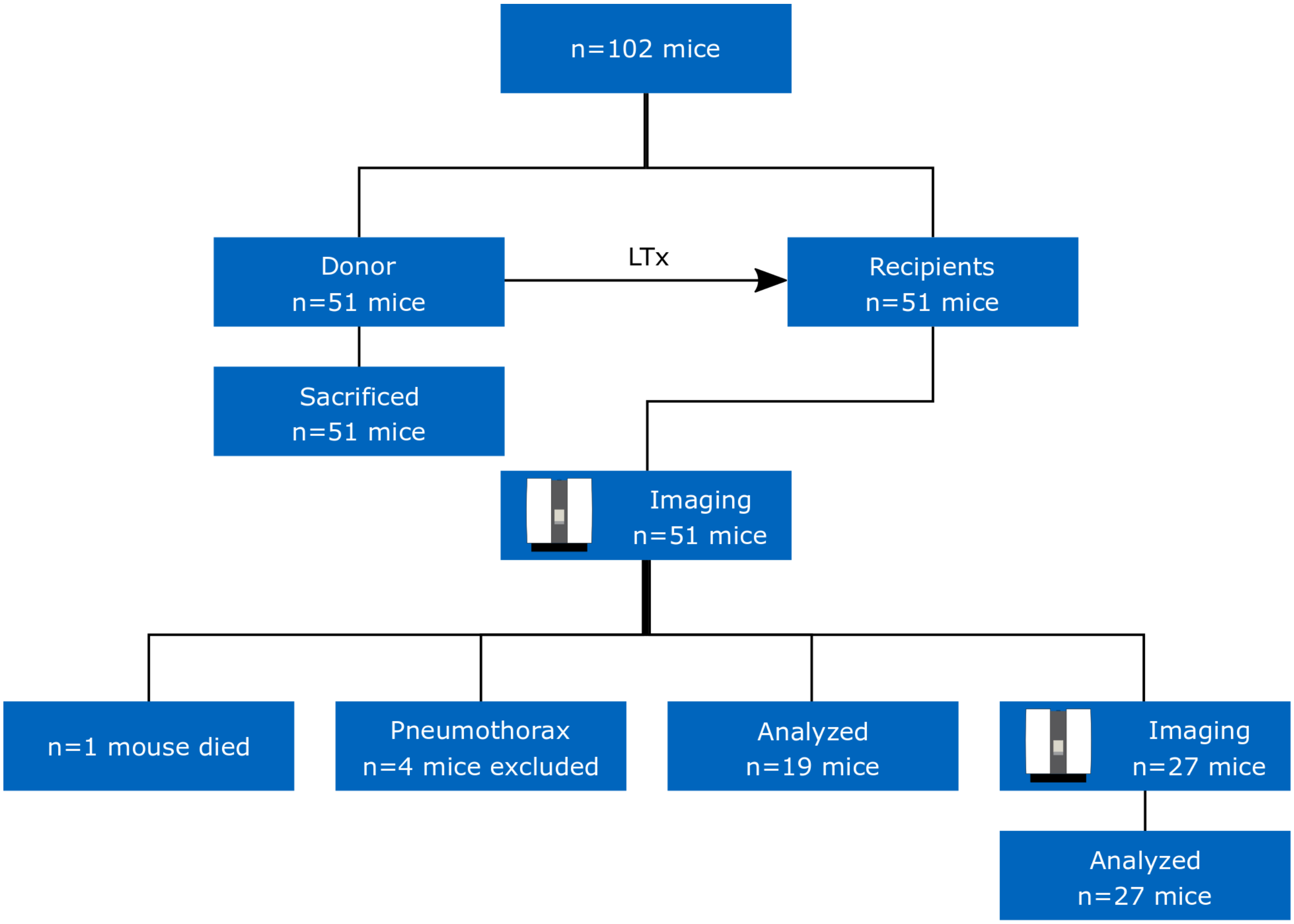

Figure 1. Scheme of the work-flow within this study.

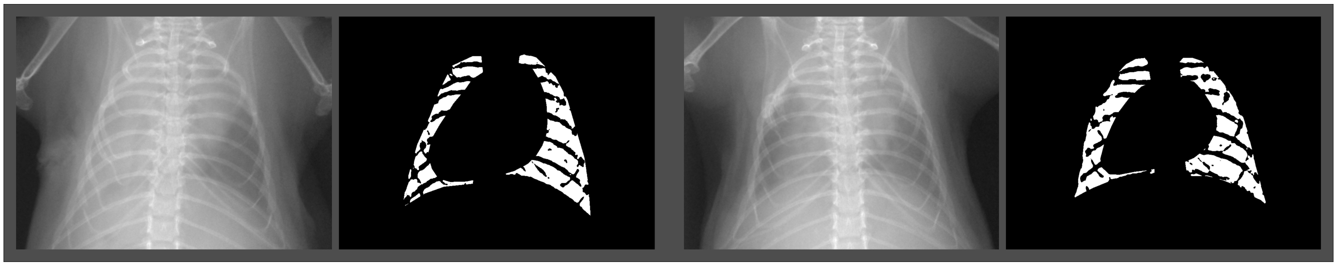

Figure 2. Example for choice of the ROIs for two transplanted mice. The segmentation was performed using MATLAB version R2018b (https://www.mathworks.com/products/new$_{p}roducts/release2018b.html$).
